# Effects of a similar amount of regular non-structured or competitive physical activity across late adulthood: a cross-sectional study

**DOI:** 10.3389/fspor.2024.1416080

**Published:** 2024-05-30

**Authors:** M. Palumbo, R. Modena, L. Bortolan, S. Skafidas, A. Callovini, A. Savoldelli, F. Gilli, A. Fornasiero, F. Schena, B. Pellegrini, C. Zoppirolli

**Affiliations:** ^1^ CeRiSM (Research Center Sport Mountain and Health), University of Verona and Trento, Rovereto, Italy; ^2^ Department of Neuroscience, Biomedicine and Movement Sciences, University of Verona, Verona, Italy; ^3^ Department of Engineering for Innovation Medicine, University of Verona, Verona, Italy; ^4^ Department of Cellular, Computational and Integrative Biology (CIBIO), University of Trento, Trento, Italy

**Keywords:** successful aging, health, older adults, exercise, training, master athletes

## Abstract

**Introduction:**

Master athletes are examples of successful aging. It is not clear whether it is the competitive-oriented training or just the amount of total regular exercise that reduces the age-related decline in physiological functions. We aimed to compare health-related parameters in competitive (C) and physically active older adults (A) that performed the same weekly physical activity (PA) amount.

**Methods:**

Seventeen C and 17 A were matched for age (8 and 9 male participants under and over 70 years old respectively, for both groups) and weekly PA amount (GPAQ). Body composition, leg and arm maximal strength, balance and reaction time were measured; moreover, leg and arm exercise efficiency, estimated VO_2max_, and VO_2_/HR relationships were evaluated. Perception of life and sleep quality was also assessed through specific questionnaires (SF-36 and PSQI). The effect of group (C vs. A), age (U70 vs. O70) and their interaction was examined through a Two-Way ANOVA test.

**Results:**

C dedicated more time to vigorous PA compared to A (*p* = 0.03), while less to moderate daily work (*p* < 0.01) and active commuting (*p* = 0.06). C exhibited better body composition (all *p *< 0.05), higher leg maximal strength (*p* < 0.05) and a trend for elevated arm strength (*p* = 0.06). Reaction time, leg and arm cycling efficiency were similar in the two groups (all p > 0.05), while balance reduced in A O70. Estimated VO_2max_ was higher for C in leg cycling (*p* = 0.05) and remained constant across ages (all *p* > 0.05). VO_2_/HR relationship, life and sleep quality did not differ for groups and ages.

**Conclusions:**

Regular physical exercise of about 6,000 METs/week seems to have a beneficial effect on health-related parameters, both in non-structured and competitive PA, when compared to sedentary behaviour. However, the older adults engaged in competitive training exhibit further advantages: better body composition, higher arm and leg muscle strength, and higher leg VO_2max_. This study highlights the importance of encouraging active lifestyles for maintaining long-term health, high levels of life quality perception and reducing age-related decline. However, vigorous training suitability needs to be verified by a team of PA specialists.

## Introduction

Ageing is a complex and inevitable biological process that brings several changes regarding various aspects of people's lives. It impacts everyone differently, influencing physical, cognitive, and emotional well-being. As far as concerns the physical domain there is a weakening in overall performance. The natural ageing process is related to decreased bone mineral density, increased tendon stiffness and sarcopenia, leading to heightened exposure to osteoporosis and fractures, impaired mobility, muscle mass and strength decline (1). A reduction in neuromuscular excitability is also expected when entering the later stages of life, with slower nerve signalling, impaired physical movement coordination and augmented risk of falls (2). It is suggested that throughout the ageing process, muscle strength declines more rapidly than muscle mass, likely due to the faster reduction in neuromuscular excitability (3).

In this context, proprioceptive sensation (particularly in the lower extremities), coordinative abilities and voluntary movement planning are also influenced by aging (4, 5). As an example, balance capacity decreases in older adults participants, due to heightened thresholds of vibrations' perception at the ankle joint and diminished control of rapid arm movements (4, 5). The cardiovascular system undergoes changes as well, with structural and functional alterations of heart and blood vessels (6). An increased arterial stiffness is often associated with aging, together with remodelling of cardiac chambers as well as impaired diastolic or systolic function of left ventricle (7). These alterations make older adults more susceptible to cardiovascular diseases, reducing their capacity to provide oxygen to the tissues, thus diminishing exercise tolerance (8, 9). Maximal aerobic capacity declines nonlinearly with age, by 3%–6% per decade in the third and fourth decades of life, while by more than 20% per decade after the age of 70 (8, 10).

The adaptation to life's changes is challenging for older adults, leading to possible psychological fragility like anxiety and depression and contributing to reducing life quality (11). However, it has been shown that the most important reason for physical decline is physical inactivity, which is often associated with the aging process itself. Indeed, exercise seems to play a major role in promoting health and well-being among older adults, contributing to both physical and mental vitality (12). Conducting an active lifestyle has beneficial effects on functional fitness which can positively impact the cognitive-emotional dimension of mental health (13). Engaging in regular physical activity becomes crucial for maintaining overall health in older adults, mitigating the risk of chronic pathological conditions and allowing independence in later life (12, 14, 15). 500–1,000 METs/week (with 1 MET representing the metabolic equivalent of the caloric consumption at rest) has been suggested as an amount of physical activity usually associated with substantial health benefits in older adults, and a predictor of successful aging (16, 17).

Exercise aids in preserving bone density and muscle mass, preventing the onset of conditions like sarcopenia and osteoporosis. It contributes in maintaining neuromuscular functions, coordination, and muscle strength (18). It also enhances cardiovascular health, reducing the risk of heart disease and promoting efficient circulation (19). Engaging in a physical activity regimen appears to play a role also in preserving postural control (4). It seemed that the relative rate of decline of maximal aerobic capacity is similar between sedentary and active older adults. However, the higher baseline of the trained individuals allows them to maintain a higher maximal oxygen consumption across aging (20). Beyond its physical benefits, exercise has profound implications also respect to mental health and overall life quality perception in the aging population (21).

As an example, master athletes (participants over 35 years of age who train and compete regularly, often until into their 80s or even 90s) were shown as individuals that defy the usual expectations of physical, cognitive and socio-emotional declining as they age (22, 23). Often non-elite athletes in their past, master athletes become exemplars of successful aging, displaying remarkable physical performances, functional capacity, intrinsic motivation, and positive psychological outlooks (9, 17, 20, 23). In recent years, an increasing number of master athletes was noticed in dedicated events (24). Regular and structured training in these athletes may reduce age-related decline in muscle strength and preserve cardiovascular function, attenuating the decline in VO_2max_ (20, 25–28).

Previously, focus was addressed on mental and physical effects given by competitive-oriented training only conducted by master athletes, or by time-limited and specific exercise programs performed by older adults aiming at different purposes (9, 20, 27–33). However, the impact of a considerable amount of regular non-structured physical exercise in older people has rarely been explored in the specific scientific literature, thus impeding awareness about the effect of physical exercise in non-athletes regularly active older adults. Therefore, here we proposed to evaluate the effects of comparable amounts of regular weekly non-structured exercise and competitive-focused training in two groups of male older individuals, with the aim to evaluate the health-related physical and socio-emotional parameters in physically active (A) and competitive (C) older individulas, over the course of their late adulthood. We hypothesized that C could display better health-related parameters, with A still showing good values compared to the normative data for the sedentary population, due to their regular active lifestyle. Moreover, we hypothesized that the negative effects of age on the health-related parameters should be limited in those populations. However, the paucity of data regarding regularly active older adults leaves us doubtful about the effect of aging process on this population.

## Methods

### Participants

Competitive older adults (C) were recruited within the project “Marcialonga Science 2019” with the support of the Marcialonga (a renowned 75 km cross-country skiing classic competition) Organizing Committee (https://www.marcialonga.it/DC/68833/marcialonga-science-con-il-cersim.php), by inviting all the Italian master athletes over 65 years old, participating to the last 10 editions of the race. They reported to be engaged in structured training, with 1 strength sessions per week of about 10–20 min on average, and 3 long endurance sessions per week between 1 and 2 h; some of them reported also interval training with high intensity repetitions. Active older adults (A) were recruited within the fitness project “Men in shape”, promoted by the Municipality of Rovereto, and selected among those individuals performing additional regular non-structured physical activity. The participants were fully informed about specific inclusion criteria and testing procedures and gave their written consent to participate in the investigation. This study was previously approved by the Ethical Committee of the University of Verona (prot. CARU 0480862/2018 proved on 28/10/2018). As the first exclusion criteria, they all were checked by a medical doctor to exclude cardiovascular, respiratory, metabolic, osteoarticular, neuro-degenerative, or psychiatric diseases or hospitalizations in the last two years.

A total of 41 competitive (range 65–79 years of age) and 21 active older adults (range 65–84 years of age) were then tested. Finally, only 17 male participants for each group were included in the data analysis, after having matched the participants on the base of their age and weekly physical activity amount, as assessed by GPAQ questionnaire (see further for details). For both C and A groups, 8 participants were under 70 years of age, while 9 were over 70.

### Measures and instrumentation

#### Anthropometric measurements

Anthropometric measurements were conducted with participants in minimal clothing in a quiet and comfortable room. Weight and height were measured by an expert technician, according to the ISAK protocol (34), to the nearest 0.1 kg and 0.001 m, respectively, while BMI was calculated. Skinfolds were obtained by using a Harpenden calliper on four sites: chest, midaxillary, subscapular, and thigh, employing a professional skin folder (GIMA, Metrica S.p.A., San Donato Milanese, Milan, Italy). The participant's body fat percentage was determined by using the equation developed by Williams et al. in 1992 (35).

#### Questionnaires

Participants completed the Global Physical Activity Questionnaire [GPAQ -validated Italian version 2, developed by the World Health Organization (36)]. This questionnaire assessed the weekly total physical activity, considering both work and recreational activities as well as active travels to and from places. It was administered face-to-face, due to its low validity if administered in self-completion modality among older adults (36). The GPAQ assigned 4 METs to moderate activities and 8 METs to vigorous activities in work, travel, and recreation. The total METs/week was determined by summing the scores of individual items. The “Short Form-36 health questionnaire” (SF-36 v.2) was also administered face-to-face, as allowed by the protocol, to assess life quality perception. It was originally designed to monitor physical and mental status in clinical studies (37), or exercise-based experimental trials (38): it investigates general perception of health and limitations in physical and daily activities due to health problems, body pain, general mental health (psychological stress and well-being, limitations in daily activities due to emotional problems) and vitality (energy and fatigue). Finally, the Pittsburg Sleep Quality Index (PSQI) questionnaire Italian version (39) was used to assess sleep quality through 19 items about sleep habits, such as sleep and wakefulness routines or problems for the past month. Higher scores correspond to greater sleep impairment.

#### Neuromuscular measurements

Arm and leg maximal isometric strength was determined from the average maximal force recorded during 3 maximum voluntary contractions (MVC), for both arm and leg extensor muscles respectively. Participants sat on a custom-built chair with hips and knees bent at 90°, secured by non-elastic belts. During arm MVC, they gripped a ski pole with a fixed elbow angle of 90° degrees. Participants were instructed to push as hard as possible against load cells (P155.B-S-A/1500N; Deltatech Italy Sogliano al Rubicone, Italy) positioned at the wrist or ankle of the dominant arm or leg. Prior to the strength test, they performed submaximal contractions for experimental setup familiarization. MVC was achieved gradually by extending the elbow or knee joint for 4 to 6 s. Force signals were acquired at 500 Hz using a data acquisition board (NI-DAQ-6016, National Instruments) and the maximal values from the 3 trials were considered as maximal isometric strength.

Participants were also tested for reaction time in an upright position, keeping arms relaxed with the hands folded in front of the body and with body weight evenly distributed on both lower limbs. They were asked to flex their shoulders and lift their arms as quickly as possible, maintaining folded hands, in response to a 1-s electronic auditory stimulus. The onset of muscle activation in the right anterior deltoid was detected using a surface bipolar EMG electrode (CDE®, Spes Medica, Genova, Italy), following SENIAM recommendations (40). Additionally, a 3-axial MEMS (Micro-Electro Mechanical System) technology full-scale accelerometer (ADXL326, Analog Devices, Norwood, MA, USA), fixed on the right upper arm, recorded the onset of shoulder flexion. All signals (sound, EMG, and acceleration) were recorded at 2,048 Hz using the electromyographic system (DuePro®, OT Bioelettronica, Turin, Italy). Signal onsets were automatically detected using a custom MATLAB (MATLAB 7.0, MathWorks, Inc., Natick, MA, USA) code, where EMG signal was previously band-pass filtered (20–450 Hz; 20 dB/oct), full-wave rectified, and low-pass filtered at 8 Hz (Butterworth type 4th-order digital filter) to obtain its linear envelope. The trial was repeated 3 times: total reaction time was considered from sound onset and arm flexion onset detected after the EMG onset.

Balance was tested with open eyes in a quiet room by using a custom-made stabilometric platform equipped with four load cells (mod. UMMC-200—Leane International srl—Italy), one at each corner. For both conditions, the participants were asked to remain upright as still as possible with the body evenly distributed on both feet and with their hands on the hips, 3 times for 20 s. The center of pressure displacement was recorded at 80 Hz (41) and the average ellipse area described was considered as the bipodalic balance (42).

#### Physiological measurements

Physiological measurements were taken during two tests specifically designed for the study: sub-maximal leg cycling for 5 min at 90 and 120 W; sub-maximal arm cycling for 5 min at 50 and 75 W. Cardiorespiratory variables and heart rate were continuously measured (K5, Cosmed, Rome, Italy), and a 20 µl blood sample was collected from the earlobe at the end of each trial, to measure blood lactate concentration (Biosen R-Line, EKF Diagnostics for life, Boerne, Texas, USA). The regression lines between oxygen consumption and heart rate (VO_2_/HR) were obtained for each participant and exercise mode, by considering the mean physiological values of the last 30 s of each step, with the slope of these regressions being derived.

Leg and arm cycling gross efficiency was calculated at the exercise intensity of 90 and 50 W, respectively, as the mechanical power output divided by metabolic power and expressed as a percentage. Metabolic power was determined by the contribution of aerobic and anaerobic metabolisms. Intensity-specific oxygen consumption per second was multiplied by the energetic equivalent for oxygen (20.9 kJ LO2-1) (42) and added to the anaerobic component, considered as 1 mMol increase in lactate from the basal value equivalent to 3.3 ml of oxygen consumption per minute (43). Finally, maximum oxygen uptake for leg cycling exercises was predicted by calculating the theoretical maximum individual heart rate with the Tanaka's formula (208–0.7 × age) specific for older adults (44) and using the individual VO_2_/HR relationships for leg cycling exercises. Similarly, maximum oxygen uptake for arm cycling exercises was predicted by calculating the theoretical maximum individual heart rate with the Hill's formula (220–age–10) specific for upper body exercise in older people (45) and using the individual VO_2_/HR relationships for arm cycling exercises.

### Statistical analyses

Data are presented as mean ± 1 SD for each group. Normal distribution of data was verified through a Shapiro–Wilk test. By using G-Power for statistics (version 3.1.9.4) and based on previously published data on older adults, we calculated a required sample size of 31 individuals (statistical power = 0.8; effect size = 0.6; alpha = 0.05; 4 groups of individuals). The total sample size is very close to that of this investigation (34 subjects). A two-way ANOVA test was employed to assess the effect of group (C vs. A), age (U70 vs. O70) and their interaction (group*age). A Bonferroni *post hoc* was performed to evaluate pairwise comparisons. The Cronbach' alpha was calculated for the SF-36 v2 and PSQI, to indicate the internal consistency estimation of the questionnaires (Cronbach’ alpha = 0,81 and 0,50 respectively). Statistical analyses were all performed with SPSS Software for Windows (Version 11.0; IBM, Armonk, NY, USA), with limit for statistical significance set at *P* < .05.

## Results

Anthropometric measurements are shown in Table 1. Differences between groups were observed for weight, BMI and fat mass, with C showing lower values than A; age did not affect these parameters significantly (*p* > 0.05). Age was 68 ± 2 and 67 ± 2 years old for C U70 and A U70, respectively, while 75 ± 2 and 74 ± 3 for C O70 and A O70, respectively. The amount of physical activity resulted in 5,505 ± 789 and 5,388 ± 2,684 METs per week for C U70 and A U70, respectively, while 4,433 ± 2,214 and 6,500 ± 3,161 for C O70 and A O70, respectively.

**Table 1:** The table presents the anthropometric parameters of the individuals participating in the study (mean ± SD) divided by group, competitive (C) and active (A) older adults and age (U70 and O70).

		U70	O70	Age effect (*p*)	Group effect (*p*)	Age* group effect (*p*)
Height (m)	C	169 ± 8	172 ± 6	.977	.489	.311
	A	174 ± 7	171 ± 6			
Weight (kg)	C	70.9 ± 10.3	70.8 ± 5.4	.575	.042	.583
	A	79.9 ± 12.5	77.4 ± 12.3			
BMI (kg/m^2^)	C	24.5 ± 1.5	24.0 ± 2.1	.541	.033	.963
	A	26.4 ± 3.0	26.3 ± 3.5			
Fat mass (% body weight)	C	18.6 ± 3.0	20.0 ± 1.9	.615	.002	.634
	A	24.0 ± 6.1	24.1 ± 4.4			

Age, group and age*group effects are indicated for each parameter.

SD, standard deviation; BMI, body mass index; m, meters; kg, kilograms.

### Questionnaires

A significant group effect was found for the item “Activity at work at a moderate intensity” with A having higher values than C (*p* = 0.006) and for the item “Recreational activities at vigorous intensity” where C displayed higher values than A (*p* = 0.029) (Table 2). Meanwhile, A showed a higher amount of “Travel to and from places”, with a trend toward significance (*p* = 0.061). Active travel significantly contributes to determining the weekly amount of physical activity in A population, accounting for 23% of the total physical weekly activity. Physical activity was maintained when passing from U70 to O70, since age did not impact any of the GPAQ scores, nor for the total or for the different items (*p* > 0.05). Moreover, a trend towards significance was found in the age*activity interaction effect for both “Travel to and from places” and “Moderate recreational activity”, where C U70 had higher values than C O70, while an opposite trend was observed for A. When considering the SF-36 questionnaire, no group or age effect (all *p* > 0.05) was found for total health score or for physical and mental components. The same was also for the sleep quality assessed through the PSQI.

**Table 2:** The table presents the questionnaire (GPAQ, SF-36 and PSQI) scores (mean ± SD) divided by group, competitive (C) and active (A) older adults and age (U70 and O70).

			U70	O70	Age effect (*p*)	Group effect (*p*)	Age* group effect (*p*)
GPAQ	Total physical activity (METs/week)	C	5,505 ± 789	5,388 ± 2,684	.570	.127	.360
		A	5,388 ± 2,684	6,500 ± 3,161			
	Vigouros work (METs/week)	C	120 ± 339	0 ± 0	.561	.397	.823
		A	160 ± 480	80 ± 240			
	Moderate work (METs/week)	C	810 ± 1,308	3,180 ± 2,436	.831	.006	.417
		A	1,307 ± 1,792	2,635 ± 2,252			
	Travel to and from places (METs/week)	C	1,080 ± 918	660 ± 561	.405	.061	.071
		A	1,045 ± 1,236	1,710 ± 965			
	Vigouros recreational activity (METs/week)	C	1,920 ± 1,643	570 ± 811	.663	.029	.831
		A	570 ± 1,019	336 ± 630			
	Moderate recreational activity (METs/week)	C	1,575 ± 1,311	593 ± 522	.158	.874	.080
		A	1,409 ± 851	1,739 ± 1,265			
SF-36	Physical health (% points)	C	85 ± 11	80 ± 10	.862	.904	.215
		A	79 ± 16	86 ± 7			
	Mental health (% points)	C	81 ± 12	75 ± 12	,643	,623	,531
		A	79 ± 19	82 ± 15			
	Total health (% points)	C	85 ± 10	79 ± 11	.752	.841	.293
		A	80 ± 19	86 ± 9			
PSQI	Sleep quality (points)	C	3.6 ± 2.6	3.9 ± 2.7	.356	.937	.900
		A	4.6 ± 2.1	4.1 ± 2.7			

Age, group and age*group effects are indicated for each parameter.

SD, standard deviation; GPAQ, Global Physical Activity Questionnaire; SF-36, Short Form Health Survey 36; PSQI, Pittsburgh Sleep Quality Index; MET, metabolic equivalent.

### Neuromuscular evaluations

#### Strength

**Figure 1:**
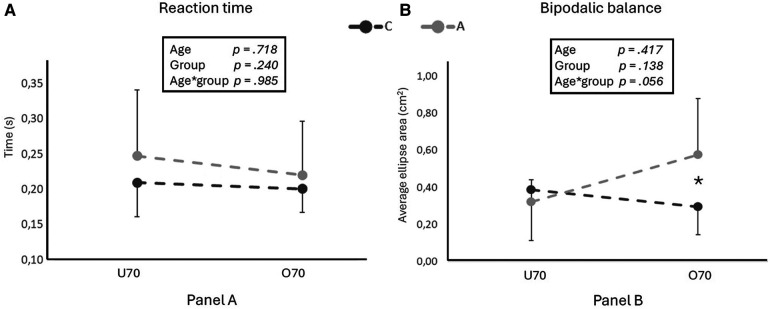
The figure shows maximal strength values for lower (**A**) and upper (**B**) limbs for competitive (C) and active (A) older adults in two different age groups (U70 and O70). In the box, age, group and age*group effects are indicated. * and ** indicate significant differences (*p* < 0.05 and <0.01, respectively) for pairwise comparison evaluated with the Bonferroni *post hoc*, when age*group effect resulted significant.

Maximal voluntary isometric contraction values resulted significantly higher in C than in A for lower limbs (*p* < 0.01; Figure 1A), while a trend toward significance was observed for upper limbs (*p* = 0.061; Figure 1B). No effect of age was detected for both lower and upper limbs (all *p* > 0.05).

#### Reaction time and balance

**Figure 2:**
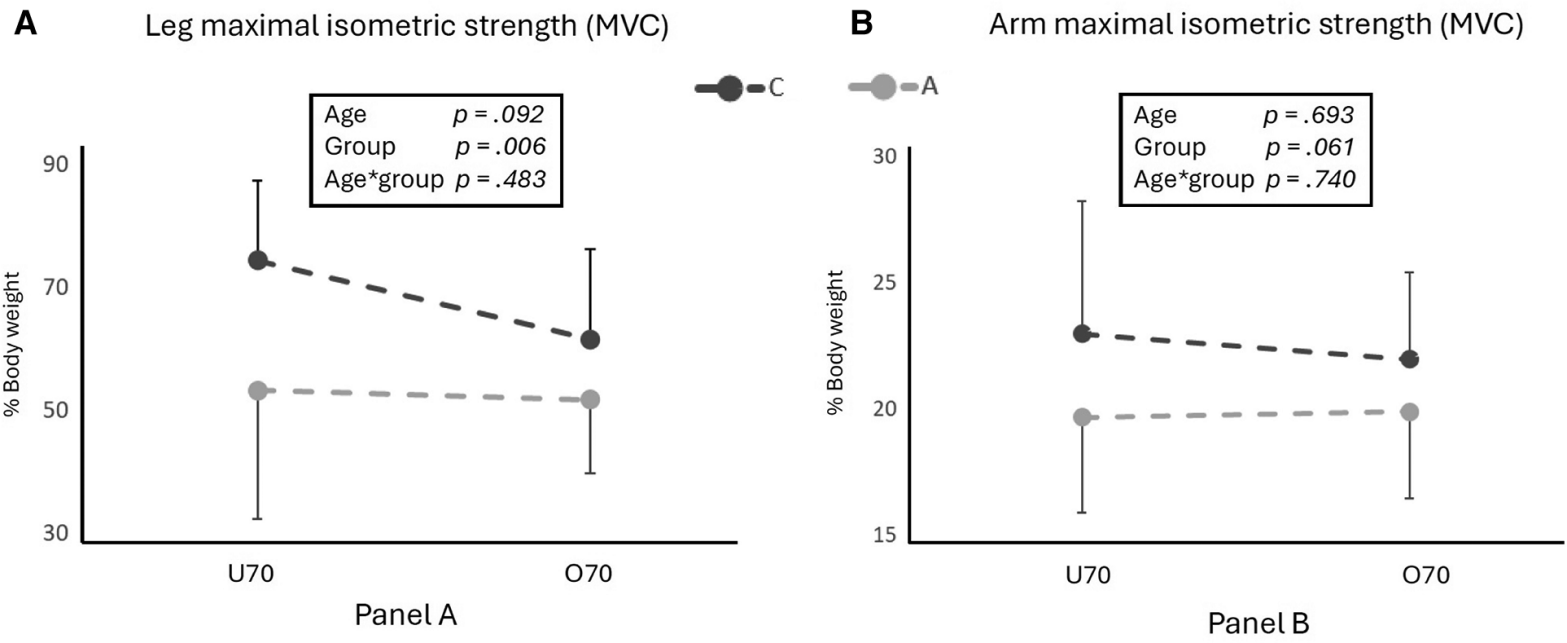
The figure shows reaction time (**A**) and bipodalic balance values (**B**) for competitive (C) and active (A) older adults in two different age groups (U70 and O70). In the box, age, group and age*group effects are indicated. * and ** indicate significant differences (*p* < 0.05 and <0.01, respectively) for pairwise comparison evaluated with the Bonferroni *post hoc*, when age*group effect resulted significant.

Reaction time to auditory stimulus resulted similar in the two groups and across age (*p* > 0.05; Figure 2A). Bipodalic balance showed a significant group*age interaction effect (*p* = 0.050), with A showing an increasing value of centre of pressure area when passing from U70 to O70 while it remained similar in C.

### Physiological measurements

**Figure 3:**
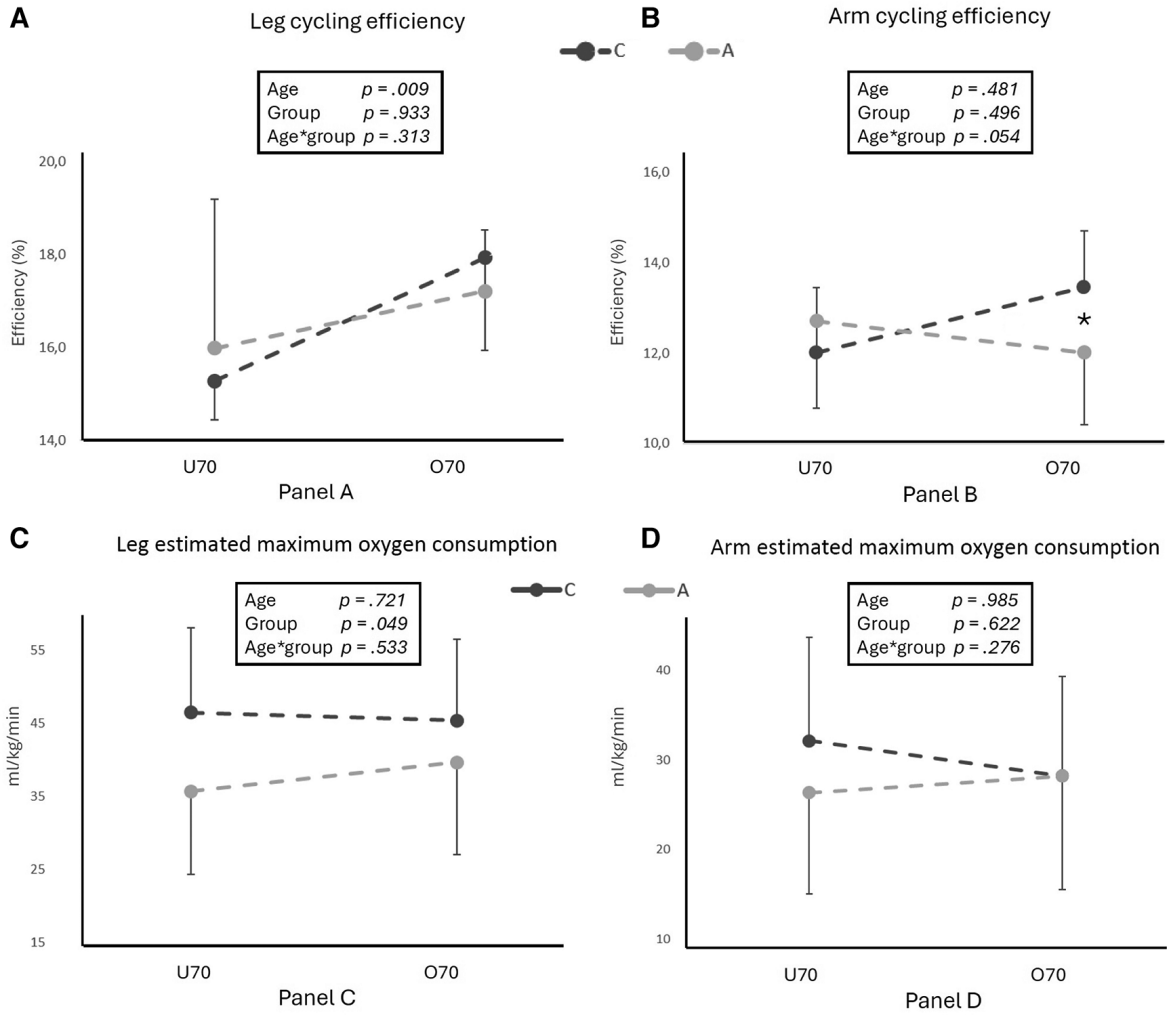
The figure shows leg cycling efficiency (**A**), arm cycling efficiency (**B**), leg estimated maximum oxygen consumption (**C**) and arm estimated maximum oxygen consumption (**D**), all for competitive (C) and active (A) older adults in two different age groups (U70 and O70). In the box, age, group and age*group effects are indicated. * and ** indicate significant differences (*p* < 0.05 and <0.01, respectively) for pairwise comparison evaluated with the Bonferroni *post hoc*, when age*group effect resulted significant.

Efficiencies for upper and lower limbs are shown in Figure 3.

Leg cycling efficiency did not differ between C and A when measured at 90 W (*p* > 0.05), but it was significantly higher when passing from U70 to O70 (*p* = 0.009). On the other hand, a significant age*activity interaction was found for arm cycling efficiency (*p* = 0.05), with C tending to increase arm efficiency with age while A showing similar values.

The slope of the equation describing the VO_2_/HR relationship for lower and upper limbs exercise did not show any group or age effect (*p* > 0.05; Table 3). We found a significantly higher estimated maximum oxygen consumption in C than in A when leg cycling (46 ± 11 vs. 38 ± 12 ml/kg/min *p* = 0.05) as in Figure 3, but not when arm cycling (*p *= 0.622). Leg and arm maximal estimated oxygen consumption remained similar across ages (*p* = 0.721 and *p* = 0.985).

**Table 3:** The table shows the slope values of the VO_2_/HR regression line equations (mean ± SD) divided by group (C and A) and age (U70 and O70).

			U70	O70	Age effect (*p*)	Group effect (*p*)	Age*group effect (*p)*
VO_2_/HR	Legs	C	0.4 ± 0.2	0.4 ± 0.2	.388	.246	.545
		A	0.3 ± 0.1	0.4 ± 0.2			
	Arms	C	0.3 ± 0.0	0.3 ± 0.2	.223	.575	.113
		A	0.3 ± 0.1	0.4 ± 0.1			

Age, group and age*group effects are indicated for each parameter.

SD, standard deviation; VO_2_, oxygen uptake; HR, heart rate.

## Discussion

The aim of our study was to assess how different physical activity patterns (i.e., regular non-structured physical activity vs. structured physical activity for competition) can impact the physical and psychological well-being of older adults as they age. Although completing a similar amount of weekly physical activity (i.e., around 6,000 METs per week), A allocated more time to moderate daily work and active commuting compared to C, while this last group dedicated more time to vigorous physical activity. Both groups exhibited positive scores of perceived health, sleep quality, body composition, lower- and upper-body strength as well as neural responsiveness, when compared with their age norms in the sedentary population. BMI and fat percentage resulted significantly lower in C than in A; C reported higher leg maximal strength and a tendency for higher arm strength compared to A. Reaction time and balance abilities were comparable between the groups, but structured training for competition purposes appeared to better preserve balance with age (i.e., O70). Leg cycling efficiency was shown to increase over time in both groups, while arm cycling efficiency was improved in C. Estimated VO_2max_ was higher for C when leg cycling (*p* = 0.05) while it was similar between the groups when arm cycling; however, both these values were maintained when passing from U70 to O70.

### Anthropometric parameters

A showed a higher body mass at similar body height, higher BMI and increased percentage of body fat mass than C. BMI is a modifiable risk factor associated with several chronic diseases and conditions. We found a significant difference (*p* = 0.033) between competitive and active older adults, in accordance with several previous studies on master athletes (32, 46). However, the mean BMI scores of both groups can be placed at the lowest point of the *U*-shaped curve designed by Winters and colleagues, indicating reduced mortality and lower risk of cardio-metabolic diseases like type 2 diabetes, stroke, and hypertension, as well as conditions such as osteoarthritis, sleep apnea, and certain cancers (32, 47, 48).

Despite differences in body fat percentages among the groups, both C and A appeared to derive beneficial effects from exercise. While the C group fell within the “excellent” category for fat body mass, the A group still experienced positive outcomes from physical activity being in the “fair” range (49). We didn't detect an age effect in any of the groups; this implied that our individuals maintained a good body composition through exercise with advancing age. These findings highlighted the overall benefits of exercise across varying fitness regimes and underscored the importance of regular physical activity for health and well-being.

### Questionnaires

The total amount of weekly physical activity was similar in the two groups, and higher compared to the world health organization recommendations (150–300 min of moderate-intensity aerobic physical activity or 75–150 min of vigorous-intensity aerobic physical activity per week, corresponding to about 600–1,200 METs per week (14). However, we found specific variations in some sections of the questionnaire. As expected, C declared to sustain more vigorous-intensity sports, fitness, or recreational (leisure) activities than their counterpart. In contrast, similar amounts of moderate-intensity activities were found in the two groups. A declared to sustain more physical activity during moderate intensity works, paid or unpaid (household chores, gardening, …), and seemed more likely to avoid the car in the everyday travels. In conclusion, while the total amount of weekly physical activity was similar in the two groups, A seemed to exploit various daily situations to perform light to moderate physical activity (i.e., cycling or walking to the shops, gardening…), while C mostly prefer to sustain their physical activity in specific exercise training, that represented their major physical task of the day. Adopting a 24-h approach in evaluating the weekly level of physical activity allows to consider both the time dedicated to recreational physical activities and what is part of daily life, including work and commuting. This is important also to develop tailored interventions and for devising strategies to ensure that movement becomes a lifestyle, especially as individuals age (50).

Data from SF-36 suggested comparable general perception of health, general mental health and vitality between groups and aligned with normative data for the general population (51). They highlighted a consistent standard of well-being in both the groups. Moreover, the PSQI results were similar, with scores <5 for both groups, indicating a “Good sleep quality” and suggesting that participation in physical activity, irrespective of age, promotes comparable health outcomes and quality of life (52).

### Neuromuscular evaluations

Knee extensor maximal muscle strength was different between groups (*p* < 0.01), even though always higher respect to the normative data for sedentary peers (53). This implies that both non-structured exercise and competitive-focused training may contribute to increase lower limb MVC capabilities beyond what is commonly observed in the older population. Upper limb extensors maximal muscle strength showed a trend toward significance (*p* = 0.060) between activity groups, with A values closely aligning with relative-aged normative, while C values being slightly higher, suggesting a potential enhancement in upper limb strength with structured training. The specific demands of cross-country skiing, which heavily involves the upper body, may have contributed to this observation. However, muscle strength seemed not to decline along different ages as usually would happen without exercise interventions. Therefore, maintaining or increasing muscle mass alone does not suffice to prevent age-related strength declines (3). In the strategies for maintaining strength while aging it is important to aim at preserving both muscle mass and neural qualities.

Reaction times appeared similar across groups (*p* > 0.05) and seemed to be lower than those proposed by Yan for the general population (4). Adopting an active lifestyle, whether through non-structured or structured physical activity, appears to positively impact neural responsiveness in older adults. It was previously suggested that regular exercise helps in maintaining neural connections and processing speed at a good level, contributing to quick stimuli' responses, even while aging (54). Also, bipodal balance was similar between the two groups and across different ages (all *p* > 0.05; Graph 2), indicating comparable balance abilities regardless of exercise regimen or age; our participants showed similar values as those reported by Daniel in a group of women who followed a physical activity program (55). However, a significant group*age interaction effect (*p* = 0.05) was observed, indicating a worsening balance for A over 70 years of age compared to stable values in C counterpart. Competitive oriented training in a discipline which requires certain balance abilities such as in cross-country skiing, seems to have a favourable impact on balance; allowing older athletes to maintain it as they age. Therefore, it seems that a high amount of physical activity (6,000 METs per week) has beneficial effects on reaction times in older adults.

### Physiological measurements

We found a significant increase in leg cycling efficiency with advancing age: in fact, U70 displayed lower efficiencies than O70, regardless of their activity group. In the dedicated literature, there is not a clear consensus on the influence of training status and age on cycling efficiency. In contrast to our results, Brisswater and Peiffer investigated different aged groups of trained triathletes, and they both found a lower cycling efficiency with advancing age (56, 57). However, Peiffer's oldest group was younger than ours (59.8 ± 1.3 years) and Brisswalter found a lowered cycling efficiency beyond 50 years with no further decrease from over 60. In his review, Tanaka asserted that exercise economy doesn't change with advancing age (9). On the other hand, our results are in accordance with Venturelli, as they also found an increased cycling efficiency with advancing age which appears to be linked to peripheral changes involving a shift towards type I muscle fiber and higher mitochondrial content (58).

Interestingly we found an age*activity interaction effect in arm efficiency, with C tending to increase also arm efficiency with age. The great involvement of the upper body in cross country skiing could have had a role in maintaining a high rate of oxygen utilization also by the arm muscles. Venturelli and colleagues found unchanged arm efficiency between young, old mobile and old immobile people during elbow flection-extension repeated exercises (58). Therefore, we can suppose that the whole-body training typical of cross-country skiers may positively influence the efficiency of upper limb function. Although Janssen et al. reported that lower limbs suffer more the advancing ageing than upper limbs, with a higher loss in skeletal muscle mass, it seems that habitual physical exercise may play a role in preserving efficiency and muscle functioning with advancing age (59). In our study we found a similar incline in both groups for the VO_2_/HR relationship when exercising with both legs and arms, indicating a similar rate of oxygen utilization at certain heart rates. Additionally, we observed a significative higher estimated maximal oxygen consumption during leg exercise, in accordance with previous studies on master athletes, without declining with advancing age (9).

### Sport, exercise and healthy aging

In the present study, we examined how different exercise patterns affect the physical and psychological well-being of older individuals as they age. Compared to the World Health Organization guidelines and previous research, our participants were doing around 5 times more volume of recommended physical activity (around 6,000 METs per week) (60, 61). Overall, we found that both structured exercise for competition and unstructured physical activity have several advantages on the health-related parameters taken into account, in comparison to the sedentary population. In accordance with previous research, engaging in regular physical activities can improve physical abilities and mental health (13, 62). Prior studies have proposed various exercise programs with both multi-dimensional and single-component approaches, with some showing greater benefits than others, but all demonstrating positive effects (29–31). With the present study, we can add that also conducting a regular active lifestyle with a high volume of self-administered physical activity has several important health-related advantages. Nevertheless, it's important to note that sports and exercise also carry risks, particularly concerning sport-related injuries, and that the suitability of high-intensity physical activity should be verified by a team of professionals specialized in human movement and sport science, like medical doctors and kinesiologists (27).

### Future perspectives

In the future, it should be interesting to include groups of older adults engaged in different sports. The distinct features of each discipline may impact differently on the health-related parameters investigated in the current study. Other endurance sports that engage mainly the upper or lower body, such as cycling, running, or rowing, could be investigated. Furthermore, situational sports like martial arts or team sports could be considered, along with disciplines requiring significant cognitive engagement like chess or those blending endurance with cognitive demands like orienteering (27, 63, 64).

### Limitations

The cross-sectional rather than longitudinal approach of this observation could be a limit. Following the same participants over time could have given us a better understanding of how exercise influences both physical and emotional health as people age. Moreover, an experimental study design would give a different insight into the effects of exercise patterns on physical and psychological well-being. In the present study, we included only male participants, potentially limiting the generalizability of the findings to the female population. This choice was due to the paucity of master female athletes in the context of cross-country skiing. Another constraint is that the VO_2max_ was estimated rather than directly measured, which could introduce some degree of error into the results. These factors should be considered when interpreting the outcomes of the research.

## Conclusion

Maintaining a consistent regimen of regular physical exercise of approximately 6,000 METs/week has shown to positively influence a multitude of health-related parameters among older adults, compared to what is known for sedentary behaviours. Notably, both non-structured exercise and competitive-focused training contributed to this favourable outcome for BMI, percentages of fat mass, lower limbs strength, reaction times and estimated maximum oxygen consumption values. Our study highlighted distinct further advantages for the older adults engaged in competitive-focused training, particularly those with intense endurance sessions and a whole-body involvement, such as cross-country skiing. These participants have exhibited enhancements across health indicators such as even better body composition, leg muscle strength, exercise efficiency when using the arms and estimated maximal oxygen consumption when exercising with the legs. Engaging in competitive sports can have further health advantages. However, the suitability of high vigorous physical activity should be verified by a team of professionals specialized in human movement and sport science, like medical doctors and kinesiologists, who could plan tailored training programs for this special population. These findings underscored the importance of promoting an active lifestyle, not only for immediate health benefits but also to reach high levels of perceived life quality, mitigating the typical decline associated with aging. We suggest that recognizing the influence of daily routines on overall activity levels is crucial for assessing the level of daily physical activity in older individuals, and eventually designing interventions to effectively encourage and facilitate regular physical activity across diverse settings and populations.

### Final consideration on master athletes

Ultimately, we demonstrated numerous benefits associated with consistent physical activity, which may be translated to good health and life quality, as well as maintenance of independence. They are particularly notable for master athletes participating in structured training. It is rare for them to have been elite in their past, implying that everyone can become a master athlete with time and passion dedicated to physical exercise; it is never too late to start practicing sport. However, considering the increased cardiovascular risk due to intense exercise in older adults, it is necessary to undergo thorough a medical evaluation before engaging in high-intensity physical activity. It is interesting that among the 4,590 male athletes taking part in Marcialonga 2019, just 417 of them were under 29 years old, ∼76% were male master skiers (over 40) and ∼20% (904) were over 60 https://www.marcialonga.it/. This means that they were able to ski for 75 km with race times ranging from a little bit less than 3 h to nearly 10 h, a notable achievement.

## Data Availability

The raw data supporting the conclusions of this article will be made available by the authors, without undue reservation.
